# Epidemiological Features of Pediatric Ocular Trauma in Egypt

**DOI:** 10.1155/2016/7874084

**Published:** 2016-10-05

**Authors:** Ebrahim Abdullah Yehia Al Wadeai, Amr Abdellatif Osman, Tamer A. Macky, Mahmoud M. Soliman

**Affiliations:** Department of Ophthalmology, Kasr El Aini Hospital, Cairo University, El-Manial, Cairo, Egypt

## Abstract

*Purpose.* To review the epidemiology of serious pediatric ocular trauma presenting to Kasr El Aini Hospital, Cairo University.* Methods.* Children with serious ocular trauma during a six-month period were examined and their data was analyzed.* Results.* Eighty eyes of 75 patients were included in this study, with 64% males (*P* < 0.001) and average age of 5 years (5 months–15 years). There were 67 (83.75%) open globe injuries, 11 (13.75%) closed globe injuries, and 2 (2.5%) chemical injuries. Of the open globe injuries, 24 (30%) were ruptured globes and 43 (53.75%) were lacerations (31 penetrating injuries (38.75%), 6 IOFBs (7.5%), and 6 perforating injuries (7.5%)). Of the closed globe injuries, 3 had hyphema (3.75%), 5 had traumatic cataracts (6.25%), and 3 had vitreous hemorrhage with retinal detachment (3.75%). Forty-two patients (56%) presented within 24 hours, 28 patients (37.33%) presented between 24 hours and 1 week, and 5 patients (6.6%) presented after one week from the time of trauma. Seven eyes developed posttraumatic endophthalmitis (10% of open globe injuries). On leaving the hospital, 55 (68.75%) eyes had poor vision, 13 (16.25%) had moderate vision, and 12 (15%) had good vision.* Conclusion.* Children at a higher risk of trauma are males, >5 years, unsupervised, and involved in street activities. Immediate comprehensive primary management and secondary rehabilitation are mandatory in these cases.

## 1. Introduction

The burden of eye trauma on societies is well documented [1–11]. Visual impairments as a result of ocular trauma lead to a variety of socioeconomic problems and today still remain a significant cause of ocular morbidity, and this impact could be devastating in children [12–23]. Ocular trauma is an important cause of ocular morbidity in children, being a leading cause of noncongenital unilateral blindness. Pediatric ocular injuries are usually uniocular and accidental but most of ocular traumas in children are preventable [23]. Pediatric ocular trauma forms about 20–50% of all eye injuries reported [22, 24–29].

Only few studies have been reported on ocular trauma in Egypt [22, 30] and especially pediatric groups [31]. Since the 2011 Egyptian revolution the pattern and rate of ocular trauma have changed [30]. This study investigates the demographics, etiology, prognostic factors, management, and visual outcomes of pediatric eye trauma at a major university hospital during a 6-month period.

## 2. Patients and Methods

Approval of the study was obtained from the hospital's ethical committee. The study design and methodology followed the tenets of Declaration of Helsinki. All patients and their parents were provided with written and signed informed consent and received a thorough explanation of the study design and aims.

This is a prospective noncomparative observational analytic clinical trial. We included children under the age of 16 years who needed hospitalization for the management of severe eye injuries. Recruitment at Kasr El Aini Hospital emergency unit started in January 2014 and was completed by the end of June 2013. We included patients referred from other departments within the hospital and referred patients from other hospitals within Cairo metropolitan area as well as from outside.

*Data Collected*. The data collected was (1) patients demographics, (2) the patient's initial complaint, (3) interval between the time of trauma and time of presentation, (4) trauma details: date, time, location, type, and mechanism, (5) clinical examination findings of the injured eye, the visual acuity, and globe injury according to the standardized classification of ocular trauma [7, 8], (6) use of eye protections, and (7) the condition of the fellow eye as a control of the visual outcome. This is done by an ophthalmologist in the emergency room through clinical examination and a questionnaire.

The visual outcome before injury and following primary repair is reported and categorized into three groups as follows: Grade I = good visual outcome, 6/36 and better; Grade II = moderate visual outcome, from 6/60 to 1/60; and Grade III = poor visual outcome, less than 1/60.

### 2.1. Statistical Methods

Statistical methods data were presented in terms of mean, range, frequency, percentages, and standard deviation. Whenever required, continuous variables were compared at 95% confidence level using ANOVA test whereas categorical variables were compared at 95% confidence interval chi-square test. All data were tabulated using Microsoft Excel version 7 sheets (Microsoft Corporation, NY) and analyzed using SPSS software version 11 (USA).

## 3. Results

**Figure 1:**
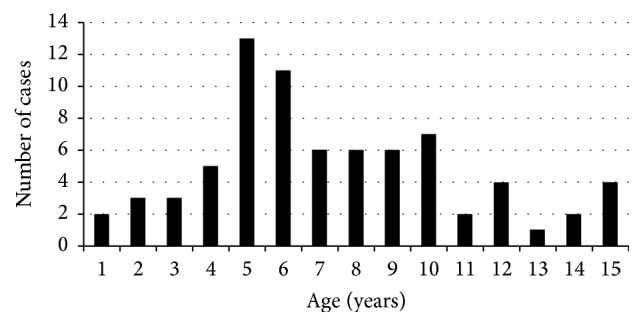
The breakdown of age in years.

In this study we included 80 eyes of seventy-five patients during the 6-month recruitment period. The mean age of the injured children in our study was 5 years (range: 5 months to 15 years). The breakdown of age and years is shown in Figure 1. Significantly more males (48 cases, 64%) were injured than females (27 cases, 36%) in our population (*P* < 0.001, chi-square test), with a male to female ratio of 2 : 1. Thirty-four cases (45.33% of all injuries) occurred at home, while forty-one (54.67% of all injuries) occurred outside home (streets). Forty-seven children (63%) were students; however, not all their injuries took place at school but mainly in neighboring areas. Twenty-six (34%) were in preschool period and two children were working (2.7%). Twenty-three injuries occurred in Cairo (30.66%), while 52 cases (69.33%) occurred outside the capital. About 70% of those that occurred in Cairo were from one area called Masr El Qadeema (Old Cairo).

Most of the injuries (30 cases; 40%) occurred during day hours especially during the period of 12:00 to 5:59 PM, and most of the patients (54 cases; 72%) presented during the same period. The majority of patients (42 cases; 56%) came to the hospital within 24 hours of their trauma. Twenty-eight patients (37.33%) showed delay of more than 24 hours to 1 week and 5 patients (6.6%) showed delay of more than 1 week.  Table 1 shows the correlations between the different governorates and the timing of injuries' presentations.

Blunt trauma occurred in 35 (44%) eyes, sharp trauma in 31 (39%) eyes, projectile injury in 12 (15%) eyes, and chemical injury in 2 (2.5%) eyes. Twelve eyes had gunshot injuries and 16 eyes had wooden sticks injuries (13 eyes during playing and 3 by assaults). Five eyes had intrastromal foreign bodies in the cornea by wooden or metal particles. Six eyes had injury by the outdoor handle or metal bar, seven by electric wire, five by toys, two by plastic pieces, four by glasses pieces on the ground, two by metal nail, three by knife, five by stones, one by pen, three by fist, and one by spoon. Trauma with sharp and blunt objects was extremely variable with no consistent instrument or mechanism (Table 2).

Eleven eyes (13.75%) had closed globe injuries, sixty-seven eyes (83.75%) had open globe injuries, and two eyes (2.5%) had chemical injuries. Of the closed globe injuries, three cases had hyphema (3.75% of all cases), five cases had traumatic cataract (6.25% of all cases), and 3 cases had vitreous hemorrhage with retinal detachment (3.75% of all cases). Of the open globe injuries, 24 eyes (30% of all eyes) had ruptured globes and 43 eyes (53.75% of all eyes) had lacerated globes, thirty-one had penetrating injuries (38.75% of all eyes), six had intraocular foreign bodies (7.5% of all eyes), and six had perforating injuries (7.5% of all eyes). Bilateral eye injuries were found in 5 patients (6% of cases). None of the children involved in ocular traumas had eyewear worn at the time of injuries. None of the injured or the fellow eyes showed previous ocular disease or previous history of ocular trauma.

The anterior segment was involved alone in 50 eyes (62.5%). Corneal and/or scleral lacerations anterior to muscle insertions occurred in 43 eyes (54%). The rest of the eyes (7) with anterior segment injuries had closed globe injuries. The crystalline lens was involved in 25% of eyes with cataract and in 5% with subluxation/dislocation. Thirty-five percent of eyes had hyphema, of which 3.75% were with closed globe injuries. The posterior segment was involved alone in 13 eyes (16.5%) of patients. Nine eyes had retinal detachment proved either clinically or by postoperative (primary repair) ocular ultrasonography. Concerning foreign bodies, we had 15 cases: 3 were in the intracorneal area, 1 was in the anterior chamber, 3 were in the midvitreal cavity, 2 were in the preretinal area, and 6 perforated the globe to settle in the orbit. Four eyes with IOFBs developed endophthalmitis following primary repair and before removal of the IOFBs.

We encountered several problems to properly measure the patients' vision on arrival. In some cases, the very young age and the magnitude of the trauma placed the children and their parents in a state of apprehension and fear of losing their sight, and therefore getting the patients' attention and cooperation was a challenge. Despite that, we were able to record visual acuity in 63 patients (67 eyes); 59 patients (88%) had poor visual acuity and 8 (12%) had moderate visual acuity.

Seven eyes developed posttraumatic endophthalmitis (10% of open globe injuries): four eyes with IOFBs, 2 with penetrating injuries, and 1 with a ruptured globe. All cases developed endophthalmitis following the primary repair between day 2 and day 10, except the ruptured globe diagnosed at the delayed presentation. Masked by the trauma, it was very difficult to diagnose endophthalmitis during the first 24 hours. None of the eyes with anterior chamber IOFBs and those with perforated injuries developed endophthalmitis. Also none of the 3 corneal foreign bodies developed infection. The 4 IOFBs eyes with endophthalmitis had their foreign bodies intravitreally and preretinally.

Intraorbital foreign bodies were found in six cases with no orbital bone fractures; all were of shotgun injuries and all six cases produced perforating globe injuries as well. Seven eyes (9.3%) had lid wounds, involving the margin in four eyes (5.3%) and the canaliculi in two cases (2.5%). There were no cases of extraocular muscles or optic nerve head avulsions reported in any of the eyes.

All patients received intravitreal and systemic antibiotics. Two patients received systemic additional antifungal on clinical suspicion of fungal infection (1 wood and 1 glass IOFBs). Two patients had a rapid deterioration of their infection with corneal infiltrations obscuring posterior segment visualization. Two patients responded well to repeated intravitreal injections and continuous intravenous antibiotics and were discharged on oral antibiotics. The remaining three had undergone vitrectomy and IOFBs removal. Unfortunately, no vitreous biopsy was taken at the time of vitrectomy. All except one had poor visual acuity on discharge.

Only 3 eyes required medical treatment alone, while the 77 eyes required surgical intervention. Forty percent of the surgical eyes required primary repair alone, in which corneal and scleral repair were 25% and 15%, respectively. However 75% of the surgical eyes required secondary intervention at a later setting. These included lens extraction and intraocular lens implantation in 24 cases (31%), vitrectomy for vitreous hemorrhage in 3 cases (4%), vitrectomy and IOFB removal in 5 cases (7%) (3 with endophthalmitis), vitrectomy for retinal detachment surgery in 9 cases (12%), and removal of hyphema in 6 cases (8%).

Patients stayed hospitalized if they chose to have their secondary procedures done in our hospital. The vitrectomies for the 3 endophthalmitis cases with IOFBs were done within 24 hours of the initial diagnosis of the infection. While these patients were prepared for surgery, the response for medical therapy is assessed and accordingly the decision for surgery is taken. All other secondary procedures were done in the second week after trauma (7–14 days).

On discharge, 55 eyes (68.75%) had poor vision, 13 eyes (16.25%) had moderate vision, and 12 eyes (15%) had good vision. Worse visual acuity occurred more with open globe injuries, ruptured globes, and those eyes that developed endophthalmitis. Twenty-eight (37%) patients were lost in followup at 1 month.

## 4. Discussion

In the current epidemiological study, we were aiming at analyzing all aspects of ocular trauma in children presented to Kasr El Aini Hospital and comparing them with other reported pediatric eye injuries. Our previous study [22] has shown a younger overall mean patients' age (22 years old) susceptible to ocular trauma in Egypt when compared to other studies by at least 10 years [5, 10, 14–16, 20]. There was also a significant increase in the pediatric portion of ocular trauma in Egypt (49.7%) compared to other studies [14, 21]. We found a higher incidence of eye injuries at the age of 5 years and above (about 65.33%), similar to what had been reported by other studies (58–60%) [23, 32–34]. Although adult supervision is essential for the safety of children, most of the injuries in our study happened in the absence of any adult presence. In this study we have found a relatively lower percentage of males (64%), compared to most of the other studies including ours [5, 14–16, 22] that had reported males to constitute about 80% of patients. Also a subanalysis of younger patients in our previous study [22] showed males to form about 74%. Indoor injuries were found to be the place of trauma in 45.5% of cases, which was also reported in other studies but with higher percentage, 50–60% [23, 32].

Our hospital received patients from all over the country, with different percentages reflecting the level of services in different regions. In this study, 61% of the patients came from Greater Cairo metropolitan area (Giza, Cairo, and Qalyubia) which is relatively lower than our previous report [22]. This may be due to an improved medical service in different governorates over the years. Most of the injuries and their presentation occurred during the period of 12:00–5:59 PM which appears to be similar to other reports including ours [21, 22]. About 43% of the patients showed a delay of presentation more than our previous study (18%) [22]. This is a very large portion, particularly if we kept in mind that these are only the patients that showed up after 24 hours and not including delays of more than 6 hours.

About 50% of patients showing delay of presentation of more than 24 hours came from areas within Greater Cairo, compared to more than 60% in our previous report mostly claiming that they did not expect the injury to be that severe requiring hospitalization. Unfortunately, few of them received improper medical and/or surgical care in nearby centers. Aghadoost et al. found that 69% of the children presented to the emergency room were within 24 hours of injury [35]. In another study by Saxena et al. in India, 24% of the patients had presented 6 hours after the injury and 34% presented more than 25 hours after injury [36].

We also found, as in our previous reports, that there was no clinically significant difference in the visual outcomes between patients presenting after 24 hours compared to those that came earlier than 24 hours. Both eyes were affected equally and bilateral injuries occurred in 6% of cases, similar to our previous study (4.1%) [22].

Trauma with blunt objects remains the major cause of ocular injuries in most of previous studies [5, 10, 14–16, 20, 21]. However, in our study there was an increase of injuries by sharp instruments as compared to other studies: blunt trauma in 44% of the eyes, sharp trauma in 39% of the eyes, projectile injury in 15% of the eyes, and chemical injury in 2.5% of the eyes. This is quiet similar to our previous report in Egypt except for the double number of projectile injuries in adults (26%). Projectile injuries constitute a significant portion of ocular trauma in Egypt. The main cause in adults [22] was during hammering, where a small piece of metal separates from an old hammer to penetrate the globe at very high speed. It contributed to a great deal of ocular trauma in workers in Egypt. Although gunshots were not a major (2%) cause 10 years ago [22], today following the 2011 Egyptian revolution gunshot injuries have increased tremendously. In this study, 12 eyes were injured by gunshot (15%). Most of shotgun injuries of the children occurred while the victims were at the balcony of their homes watching a wedding or fight in the street or while walking beside conflicts around. Aghadoost et al. found that pediatric ocular trauma most commonly occurred at home (43%) [35]. In another study conducted in Yazd, a central province in Iran, by Shoja and Miratashi [37], ocular trauma occurred most commonly at home and most of the other incidences occurred in traffic accidents.

Most common reason of admission was open globe injuries (67 eyes; 83.75%), with 43 eyes (53.75% of all eyes) having lacerated globes and thirty-one having penetrating injuries (38.75% of all eyes). Second to penetrating injuries is ruptured globes (24 eyes, 30% of all eyes) and then thirdly comes IOFB and perforating injuries (6 eyes for each, 7.5%). There were only three cases of hyphema (3.75% of all cases); five cases had traumatic cataract (6.25% of all cases) and 3 cases had vitreous hemorrhage with retinal detachment (3.75% of all cases). On the contrary, Aghadoost et al. reported that the most common cause of admission was hyphema (38.1%), followed by corneoscleral laceration (27.5%) [35]. In a large study on pediatric ocular trauma in the USA by Brophy et al. in 2006, the major type of ocular injury leading to hospitalization was hyphema [38]. In another study by MacEwen et al., 60% of the children hospitalized for ocular trauma had hyphema [23]. Penetrating and ocular trauma were the second most common cause of admission in their study, as in other researches [28, 37–39].

Anterior segment injuries alone occurred in 62.5% of cases which is less than our previous studies (72%) while posterior segment injuries increased to 37.5% of cases from 23% previously [22]. Posterior segment involvement alone was negligible in our previous report, compared to 16.5% in this study. Corneal lacerations (54%) were 3 times more than sclera lacerations (16%). Endophthalmitis appeared to occur more with lacerated globes than with ruptured globes: 57% with IOFBs, 29% with penetrating injuries, and 15% with ruptured globes, almost similar to our previous results (60%, 30%, and 10%, resp.) [22]. Despite intravitreal and intravenous antibiotics, the prognosis of posttraumatic endophthalmitis was not good, with 6 cases (85%) having poor visual acuity on discharge and followups.

Although it was difficult to properly record the visual acuity at presentation, comparing the records of presented visual acuity with that of discharge did not show any significant deterioration. On the contrary, there were improvements in some cases, where 88% of poor vision was reduced to 68.75% and the moderate vision increased from 12% to 16.25% and the good vision increased from zero to 15%. Those with deteriorated vision are due to complications like endophthalmitis, development of cataract, or retinal detachment. In this study poor vision is the main visual outcome on discharge from the hospital. However, many patients had dramatic improvement of vision later on with followup. In a study by Aghadoost et al., the initial visual acuity of 48% of the damaged eyes of patients at the time of presentation to the ER was less than 6/60, which is similar to the observation from other studies in Iran, Singapore, and USA [37, 38, 40, 41].

Pediatric eye injury in Egypt is a major problem, accounting for the majority of cases of unilateral blindness in children. However, eye trauma and its subsequent visual disability are for the most part avoidable. This study aimed at identifying factors involved in pediatric ocular trauma in an attempt to help to reduce its prevalence in Egypt.

The children at a higher risk of trauma had been identified as males, older than 5 years, unsupervised, and involved in street activities. The risk factors can be summarized in that we have relatively violent behaviors in our streets: throwing stones, knives, sticks, and guns. Trauma patients should receive comprehensive primary management, due to the poor compliance with followup examinations and secondary rehabilitation. Parents should keep children younger than 5 years always supervised and in a safe environment, away from sharp objects.

## Figures and Tables

**Table 1:** The timing of presentation and amount of delay for each governorate.

	Time of presentation				
	All cases	<24 hrs	24–48 hrs	48 hrs–1 week	>1 week
Cairo	23	18	1	4	—
Fayoum	17	7	5	4	1
Giza	14	7	4	2	1
Beni Suef	9	4	4	1	—
Qalyubia	9	6	1	2	—
Asyut	1	—	—	—	1
Arish	1	—	1	—	—
Beheira	1	1	—	—	—

**Table 2:** Assault objects used in trauma.

Causes	Number (%)
Shotgun	12 (15%)
Wooden stick	16 (20%)
Door metallic handle/metallic bar	6 (7.5%)
Metallic wire	7 (8.75%)
Fist	3 (3.75%)
Knife	3 (3.75%)
Toys	5 (6.25%)
Stone	5 (6.25%)
Falling on the ground on particles	6 (7.5%)
Chemical injuries	2 (2.5%)
Spoon	1 (1.25%)
Pen	1 (1.25%)
Metal nail	2 (2.5%)
Superficial foreign bodies	5 (6.25%)
